# A protocol for cell therapy infusion in neonates

**DOI:** 10.1002/sctm.20-0281

**Published:** 2021-01-06

**Authors:** Elizabeth K. Baker, Euan M. Wallace, Peter G. Davis, Atul Malhotra, Susan E. Jacobs, Stuart B. Hooper, Rebecca Lim

**Affiliations:** ^1^ Newborn Research Centre Royal Women's Hospital Melbourne Victoria Australia; ^2^ Department of Obstetrics and Gynaecology University of Melbourne Victoria Australia; ^3^ The Ritchie Centre Hudson Institute of Medical Research Victoria Australia; ^4^ Department of Obstetrics and Gynaecology Monash University Victoria Australia; ^5^ Department of Paediatrics Monash University Victoria Australia

**Keywords:** bronchopulmonary dysplasia, cell‐ and tissue‐based therapy, human amnion epithelial cells, infant, premature, infusions, intravenous

## Abstract

Cell therapies for neonatal morbidities are progressing to early phase clinical trials. However, protocols for intravenous (IV) delivery of cell therapies to infants have not been evaluated. It has been assumed the cell dose prescribed is the dose delivered. Early in our clinical trial of human amnion epithelial cells (hAECs), we observed cells settling in the syringe and IV tubing used to deliver the suspension. The effect on dose delivery was unknown. We aimed to quantify this observation and determine an optimal protocol for IV delivery of hAECs to extremely preterm infants. A standard pediatric infusion protocol was modeled in the laboratory. A syringe pump delivered the hAEC suspension over 60 minutes via a pediatric blood transfusion set (200‐μm filter and 2.2 mL IV line). The infusion protocol was varied by agitation methods, IV‐line volumes (0.2‐2.2 mL), albumin concentrations (2% vs 4%), and syringe orientations (horizontal vs vertical) to assess whether these variables influenced the dose delivered. The influence of flow rate (3‐15 mL/h) was assessed after other variables were optimized. The standard infusion protocol delivered 17.6% ± 9% of the intended hAEC dose. Increasing albumin concentration to 4%, positioning the syringe and IV line vertically, and decreasing IV‐line volume to 0.6 mL delivered 99.7% ± 13% of the intended hAEC dose. Flow rate did not affect dose delivery. Cell therapy infusion protocols must be considered. We describe the refinement of a cell infusion protocol that delivers intended cell doses and could form the basis of future neonatal cell delivery protocols.

## Graphical abstract

Cell therapy is neonatal medicine's new frontier. While the challenges of translation have been much discussed, we have overlooked a simple yet fundamental hurdle; a protocol that delivers the intended cell dose intravenously to infants. Our existing protocol delivered less than 20% of the intended dose of human amnion epithelial cells. Here, we demonstrate simple measures can optimize cell dose delivery.

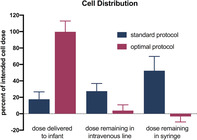

**Significance statement**

Cell therapy is neonatal medicine's new frontier. While the challenges of translating exciting preclinical discoveries have been much discussed, a simple yet fundamental hurdle has been overlooked; a protocol for the intravenous delivery of cell therapy in neonates. The validity of safety and dose escalation studies is dependent on delivering the intended cell dose. The present findings draw attention to the urgent need for evaluation of neonatal infusion protocols and provide the basis on which future protocols could be developed.

## 1 INTRODUCTION

Promising preclinical advances in regenerative cell therapies for major neonatal morbidities including bronchopulmonary dysplasia (BPD) and brain injury have led to early phase clinical trials.^1^, ^2^, ^3^, ^4^, ^5^, ^6^, ^7^, ^8^ As feasibility and early safety reports emerge, the challenges of translation have been widely documented.^6^, ^9^, ^10^ However, one challenge has been overlooked—the reliable delivery of a known dose of cells. It has been assumed that the intended dose of cells is the dose actually delivered. Such an assumption is fundamental to the validity and interpretation of early phase safety studies and of future efficacy trials. And yet that assumption has never been tested. During the early stages of our human amnion epithelial cell (hAEC) dose escalation study, we observed hAECs settling in the intravenous (IV) line and syringe during infusions given to preterm infants at high risk of BPD.^1^ This challenged the crucial assumption that infants were receiving the correct dose.

hAECs are a homogenous population of stem‐like cells isolated from the amnion, the inner of the two membranes that surround the fetus during gestation.^11^ Available in abundance from a material that would otherwise be discarded, immune‐privileged with limited expression of human leukocyte antigens class Ia and class II and nonteratogenic,^11^ hAECs are an appealing cell therapy candidate. hAECs are immunomodulatory, acting through paracrine effects to expand regulatory T cells and favor the reparative M2 macrophage phenotype.^12^ hAECs have shown therapeutic promise in preclinical models of BPD and preterm brain injury^13^, ^14^, ^15^, ^16^, ^17^, ^18^, ^19^, ^20^ and importantly, their efficacy is dose dependent.^13^ Though extrapolating from animal studies has inherent limitations, a therapeutic dose is likely in the order of 30 to 50 million hAEC/kg.^7^

Our phase 1 dose escalation study in infants at high risk of BPD^1^ begins with a dose of 2 million hAECs/kg in a single infusion escalating to 10 million hAECs/kg as the study progresses. Further dose escalation to 30 million hAECs/kg is planned by giving repeat infusions of 10 million hAECs/kg administered at 5‐ to 7‐day intervals. Recruitment commenced in 2018 and is expected to be complete during 2022. Our population, infants born at less than 29 weeks gestational age, present unique challenges. Their bodyweight, generally in the range of 500 to 1000 g, and hemodynamic immaturity necessitate small volumes infused slowly. Clearly, delivering the prescribed dose is fundamental to our dose escalation study.

In this laboratory‐based study, we aimed to quantify the hAEC dose delivered using our standard infusion protocol, then evaluate components of the cell delivery system that can influence the dose delivered. These include method of cell agitation, IV‐line volume, albumin concentration, and syringe orientation. And finally, design an infusion protocol that consistently delivers the intended cell dose.

## 2 METHODS

### 2.1 hAEC collection and isolation

Placentae from uncomplicated, term pregnancies were collected at the time of planned caesarean section following written, informed consent, and with approval from Monash Health Human Research Ethics Committee (HREC number 12223B). hAECs were isolated and stored as described previously.^21^

### 2.2 Preparation of hAEC suspension

hAECs were thawed in a water bath at 37°C, washed with 2% albumin, and centrifuged at 350*g* for 5 minutes. The hAEC pellet was resuspended in 2% albumin at a density of 1.25 million live hAECs/mL in anticipation of a 20% cell loss during filtration.^7^ Twenty milliliters of hAEC suspension was injected into a 150‐mL transfer bag (Teruflex Transfer bag, Terumo Corporation, Tokyo, Japan), then filtered through a blood transfusion set with a 200‐μm filter (Baxter Pediatric Blood Component transfusion set, Baxter Healthcare SA, Zurich, Switzerland) into a 30‐mL syringe and the IV line (volume 2.2 mL, length 150 cm) of the transfusion set primed.

### 2.3 Modeling standard hAEC infusion protocol

An infusion protocol typical of that used in neonatal practice was modeled (the “standard infusion protocol”). A syringe pump (Alaris GH Plus syringe pump, BD, Rolle, Switzerland) was programed to deliver 5 mL/h for 60 minutes, mimicking a clinically plausible dose. An anticipated 20% cell loss during filtration results in a suspension density of 1 million hAECs/mL, 5 mL/h for 60 minutes delivers 5 million hAECs, equivalent to 10 million hAECs/kg for a 500‐g infant. The infusate was collected in four 15‐minute aliquots (referred to as aliquots 1, 2, 3, and 4), and the volume of each aliquot was measured. At 5‐minute intervals the infusion was paused, the syringe removed from the syringe pump and manually agitated to resuspend the hAECs, before continuing the infusion. The hAEC suspension contained in the IV line at the end of the infusion was collected by flushing the IV line with 5 mL of 0.9% sodium chloride. The suspension remaining in the syringe at the end of the infusion was agitated to resuspend the cells and retained. The experiment was performed five times.

### 2.4 Measuring cell density

**FIGURE 1:**
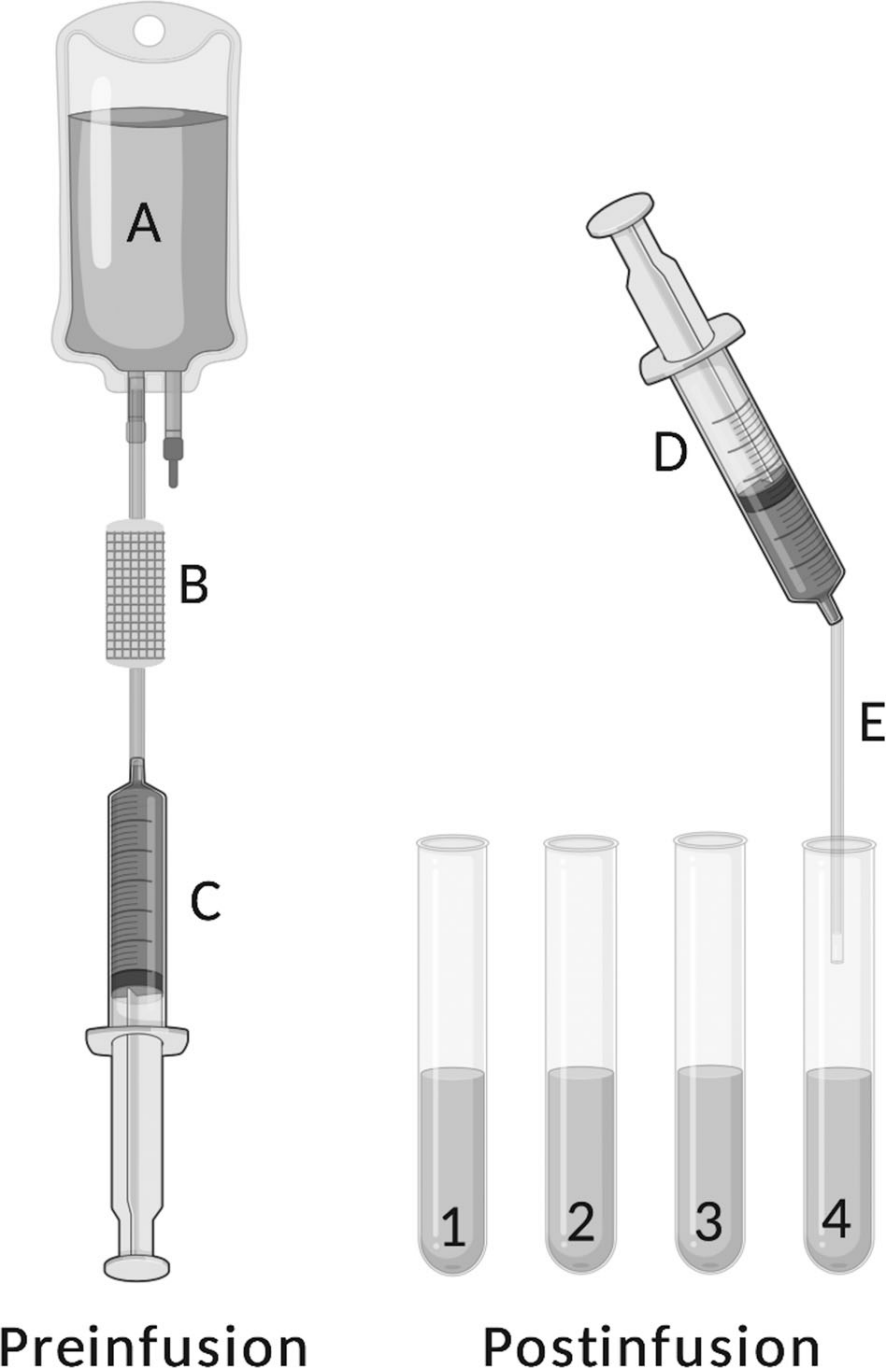
Preinfusion: the prefiltration human amnion epithelial cell (hAEC) suspension contain in the transfer bag, A, is drawn through the 200‐μm filter, B, to a syringe, C. Postinfusion: the hAEC infusate is collected in four aliquots (1‐4) during the infusion. At the end of the infusion both the syringe, D, and the intravenous (IV) line, E, contain hAEC suspension. Image created with BioRender.com

The hAEC viability and density of the suspension were measured prefiltration and postfiltration, in each of the four aliquots, the IV line, and the syringe at the end of the infusion (Figure 1). The hAEC viability and density were measured in duplicate using an automated cell counter (Countess, Thermo Fisher Scientific, Invitrogen, Scoresby, Victoria, Australia) and averaged. Viable cell counts were determined through trypan blue exclusion and are reported unless otherwise specified.

### 2.5 Calculating hAEC dose delivery

The “intended hAEC dose” was calculated using the postfilter hAEC density and the volume the syringe pump was programed to deliver. The “actual dose” delivered was calculated using the measured hAEC densities and volumes of aliquots 1, 2, 3, and 4.

### 2.6 Infusion protocol variations

Cell delivery was evaluated with the following sequence of infusion protocol variations; each experiment was repeated five times. Infusion protocols adopted the conditions determined as optimal for cell delivery by the preceding experiments.

1. A magnetic flea was placed within the syringe to allow agitation without interrupting the infusion.
2. IV lines of varying priming volumes (1.2, 0.6, and 0.2 mL) were substituted.
3. The albumin concentration was increased from 2% to 4%.
4. The syringe and IV line were positioned vertically rather than horizontally.
5. Delivery was evaluated in the absence of agitation to identify a simpler infusion protocol for the clinical setting.
6. Finally, flow rate (3‐15 mL/h) variation was evaluated.

As the postfilter density varied between each replication (1.19 × 10^6^ ± 2.3 × 10^5^ hAEC/mL), hAEC density is described as a “percentage of hAEC starting density” (ie, percentage of the hAEC postfilter density). Similarly, the infusate volume and hAEC dose delivered are described as a percentage of the “intended” volume and hAEC dose, respectively.

### 2.7 Statistical analysis

Data for each experimental group are expressed as mean ± SD. Differences between two experimental groups were determined using unpaired two‐tailed *t* test. Differences across three or more groups were determined using one‐way analysis of variance with the Bonferroni post hoc test. Confidence intervals of 95% were deemed significant.

## 3 RESULTS

### 3.1 Standard delivery protocol

**FIGURE 2:**
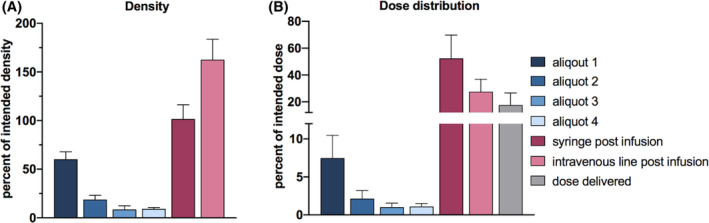
Cell density and dose distribution using the standard infusion protocol. A, Density of the human amnion epithelial cell (hAEC) suspension expressed as a percentage of the starting hAEC density. B, The dose distribution at the end of the infusion. Aliquots 1–4: four 15‐minute aliquots collected during the 60‐minute infusion. Syringe postinfusion: suspension remaining in the syringe at the end of the infusion. Intravenous (IV)‐line postinfusion: suspension collected from the IV line at the end of the infusion. Dose delivered: percentage of intended dose delivered to the “infant,” where the intended dose was calculated using the intended volume and starting cell density

The actual cell dose delivered using the standard protocol was 17.6% ± 9% of the intended cell dose (Figure 2B). The reduced dose was in part due to the progressive decrease in hAEC density in aliquots 1 to 4 (Figure 2A). The hAEC density of aliquots 3 and 4 was less than 10% of the starting density. hAECs accumulated in the IV line such that the cell density in the IV‐line postinfusion was 163% ± 21% of the starting hAEC density resulting in 27.5% ± 9% of the intended dose remaining in the IV line (Figure 2).

**FIGURE 3:**
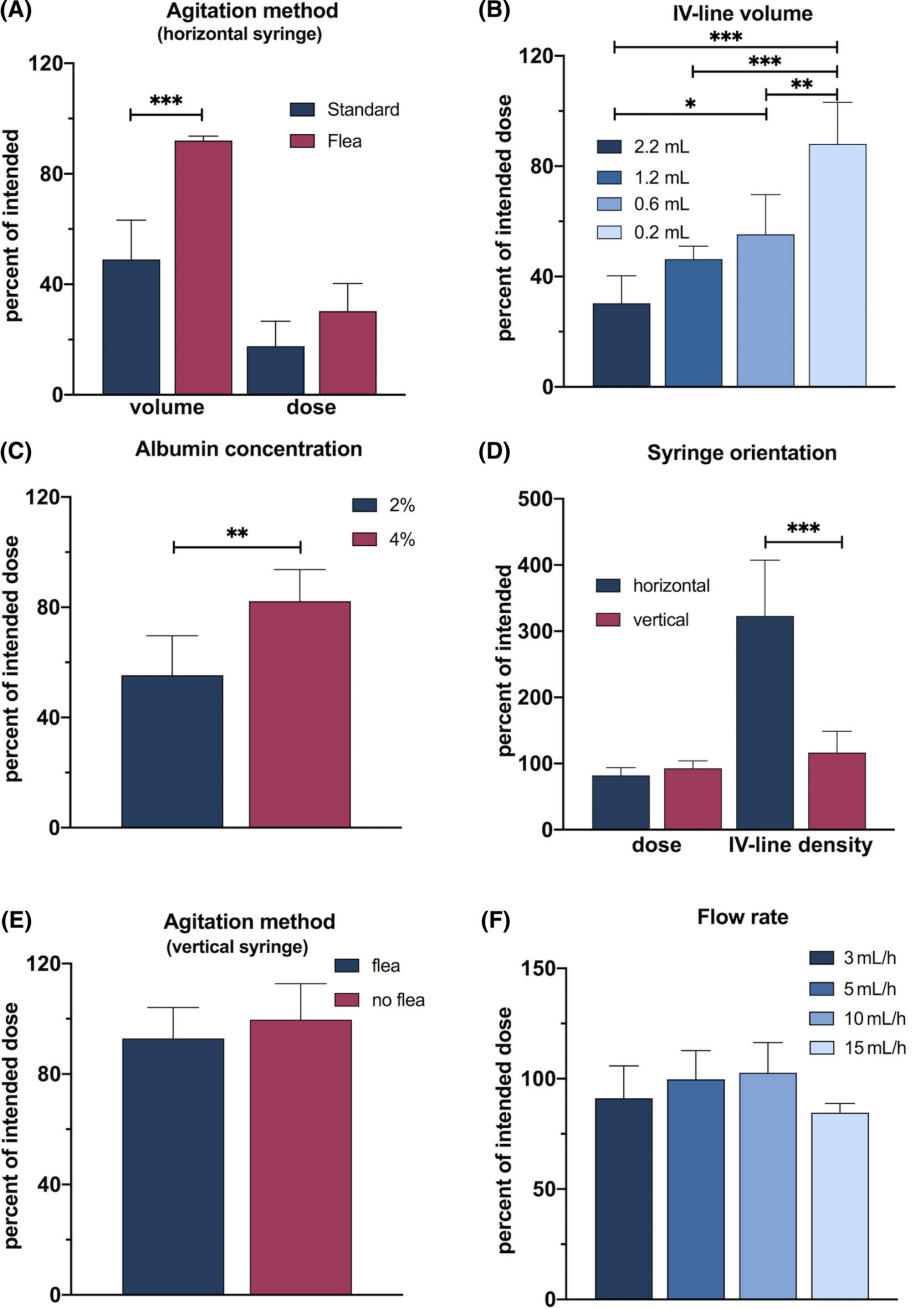
The effect of protocol variations on human amnion epithelial cell (hAEC) dose delivery. Volume and dose delivered with variations in mode of agitation, A. Dose delivered with variation in intravenous (IV)‐line volume, B, albumin concentration, C, and agitation method (vertical syringe), E. Dose delivered and IV‐line hAEC density with variation in syringe and IV‐line orientation, D. Dose delivered with variation in flow rate, F. Significance bars: *P < .05, **P < .01, ***P < .001

A second factor contributed to the decrease in cell dose delivered; the volume of suspension delivered was 49% ± 14% (2.45 ± 0.7 mL) of the intended 5 mL (Figure 3A). We observed that pausing the infusion and regularly removing the syringe from the pump interrupted the pump function. The pump took some time to reestablish the flow rate after the syringe was replaced. This phenomenon, common in syringe pumps and referred to as the “start up trend,” likely accounted for the decreased volume delivered.^22^ As a result, 52.4% ± 17% of the intended dose remained in the syringe (Figure 2B).

### 3.2 Alternate methods of agitation

A magnetic flea within the syringe allowed agitation with an external magnet, negating the need to pause the infusion and remove the syringe, restored the volume delivered (Figure 3A). This method of agitation delivered 92% ± 1.6% of the intended volume compared with the 49% ± 14.2% delivered by the standard protocol (*P* = .0001). However, despite the improved volume delivered, the actual cell dose delivered increased only modestly compared with the standard protocol (30.3% ± 10.0% vs 17.6% ± 9.0%, respectively, *P* = .07; Figure 3A) and remained lower than intended. Agitation with a magnetic flea was incorporated for subsequent experiments, given the improved volume delivery.

### 3.3 IV‐line parameters

Using a magnetic flea to agitate the suspension, we next assessed the impact of varying the IV‐line volume on the dose of cells delivered. With decreasing IV‐line volume, the hAEC dose delivered progressively increased from 30.3% ± 10.0% for a 2.2 mL line, 46.3% ± 4.7% for a 1.2 mL line, and 55.3% ± 14.4% for a 0.6 mL line, to 88.1% ± 15.0% for a 0.2 mL line (*P* < .0001; Figure 3B).

Although the 0.2 mL IV line delivered the highest percentage of the intended cell dose, it is only 25 cm long. We considered such a line length impractical for clinical use because the intended recipients, extremely preterm infants, are in incubators. Therefore, we adopted the 0.6 mL IV line, which is 75 cm long, for all subsequent experiments.

### 3.4 Albumin concentration

Using a 0.6 mL IV line and agitating the suspension with a magnetic flea, we next assessed the effect of varying the concentration of albumin in the cell suspension vehicle. Compared with 2% albumin, suspending the hAECs in 4% albumin increased the percentage of intended hAEC dose delivered from 55.3% ± 14.4% to 82.2% ± 11.5% (*P* = .007; Figure 3C). We suspended hAECs in 4% albumin for subsequent experiments.

### 3.5 Orientation of syringe and IV line

Using a 0.6 mL IV line, agitating the suspension with a magnetic flea and suspending the cells in 4% albumin, we next assessed whether the orientation of the syringe and IV line‐horizontal or vertical‐affected cell delivery. There was no significant difference between the percentage of dose delivered via vertical and horizontal orientation (82.2% ± 11.5% vs 92.9% ± 11.2%, respectively, *P* = .15; Figure 3D). However, fewer hAECs settled in the vertical IV line than in the horizontal IV line (323% ± 83% vs 116.8% ± 32.2%, *P* < .001; Figure 3D). This observation suggests that under these conditions hAEC accumulation is likely gravity dependent rather than a result of adhesion to the IV line.

### 3.6 Cell delivery without agitation

**FIGURE 4:**
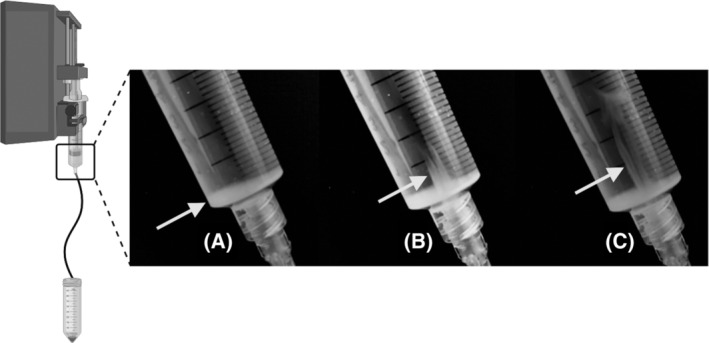
The vertical syringe and intravenous (IV)line set‐up. In the absence of agitation, human amnion epithelial cells (hAECs) settle in the syringe (A, arrow), highlighted by the plume of cell suspension that is generated when the syringe plunger is withdrawn (B,C, arrows). Image created with BioRender.com

Aware of the logistical challenges in providing cell agitation in a clinical setting, using the optimal conditions established in experiments ii to iv (0.6 mL IV line, 4% albumin and vertically orientated syringe and IV line), we reevaluated the cell dose delivered without a flea agitating the suspension. In the absence of agitation, the cell dose delivered did not significantly differ from that with agitation (99.7% ± 13% compared with 92.9% ± 11.1%, *P* = .40; Figure 3E). However, hAECs were observed to settle in the vertical syringe in a gravity‐dependent manner near its exit point (Figure 4). At the end of the infusion, the hAEC density in the syringe was 131.4% ± 43.7% of the starting density.

### 3.7 Influence of flow rate

When delivering hAECs suspended in 4% albumin, from a vertical syringe and 0.6 mL IV line with no cell agitation, altering the flow rate, 3 to 15 mL/h did not significantly alter the hAEC dose delivered (*P* = .11; Figure 3F).

### 3.8 hAEC viability

**FIGURE 5:**
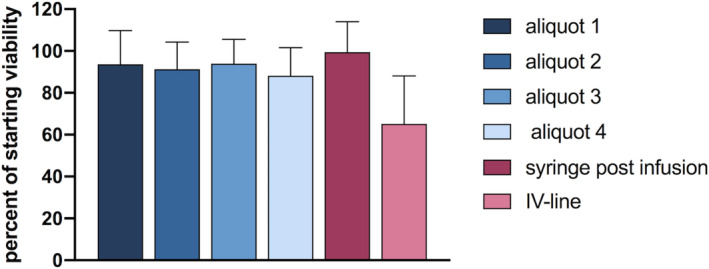
Human amnion epithelial cell (hAEC) viability. Suspension viability, expressed as a percent of starting viability, during optimal cell delivery conditions. Aliquot 1, 93.7% ± 16%; aliquot 2, 91.2% ± 13%; aliquot 3, 93.9% ± 12%; aliquot 4, 88.2% ± 13%; syringe postinfusion, 99.4% ± 15%; intravenous (IV) line, 65.1% ± 23%

During optimal cell delivery conditions, the viability of the suspension remaining in the syringe at the end of the infusion was 99.4% ± 15% of the starting (postfilter) viability. Similarly, the viability of the delivered suspension (aliquots 1‐4) was maintained (Figure 5). However, the viability of the suspension that collected in the IV line (65.1% ± 22% of the starting viability) was significantly lower than that in the syringe and aliquots 1 to 4 (*P* < .0001).

## 4 DISCUSSION

Cell therapies are an exciting prospect in neonatal medicine and the challenges of translating promising preclinical findings have deservedly been given much attention. One of the practical considerations, perhaps overlooked until now, is the challenge of delivering very small volume infusions slowly to extremely low birthweight infants.

Through a progressive experimental design, we have identified key infusion variables that significantly alter the number of cells actually delivered and, from these insights, designed a protocol for cell therapy delivery specific to the challenges of neonatology.

The sequence of protocol variations was pragmatic, designed to find simple and clinically practical solutions. We started by addressing the protocol limitations that contributed most to dose loss. Given the original protocol only delivered half the intended volume, we first sought to rectify this limitation. Then, we sought to minimize the large proportion of the dose that was lost in the IV‐line with changes to the IV‐line volume. Increased albumin concentration was used to minimize cell settling. Then, using the cell settling that remained, we explored positioning the syringe and IV line vertically.

We have demonstrated that simple measures, small‐volume IV lines, increased albumin concentration and a vertically positioned syringe and IV line improve hAEC delivery to 99% of the intended dose without physical agitation or mixing of the cells. We have also demonstrated that variations in flow rate, which may be required clinically, do not affect dose delivery when other infusion parameters are optimized.

That our standard infusion protocol was delivering less than 20% of our intended dose was disappointing to us and undermined the validity of our dose escalation study.^1^ We had completed enrollment to our first dose cohort (intended dose 2 million hAECs/kg). Upon assessment of our findings, we suspended recruitment and disclosed this finding to our enrolled subjects' families. With approval from our governing human ethics committee, and after validation of the new delivery protocol, we resumed recruitment enrolling a further three infants to repeat the 2 million hAECs/kg dose cohort. Recruitment to this dose escalation study is expected to be complete during 2022.

This study casts doubt upon the conclusions drawn from previous trials of IV cell therapy in neonates, though reports of detailed infusion protocols used during neonatal cell therapy are sparse.^7^, ^23^ Our group reported the protocol used in our first‐in‐human safety study of hAECs.^7^ Six infants with established BPD were given 1 million hAECs/kg intravenously. Five of the six infants studied received hAECs suspended in 0.9% saline, filtered using a pediatric blood component transfusion set, a standard volume IV line (2.2 mL priming volume) delivered over 30 minutes from a syringe orientated horizontally on a rocking platform. Based on our current modeling, the dose of hAECs received by these infants was likely significantly less than the reported 1 million/kg.

Tsuji and colleagues administered autologous umbilical cord blood (UCB) cells to term infants with hypoxic ischemic encephalopathy (HIE) intravenously over 60 minutes using a syringe pump with intermittent agitation to prevent cell settling. An earlier study of autologous UCB cells for late preterm to term neonates with HIE described flushing the intravascular line with 1 to 2 mL of 0.9% saline following the infusion, though the description of the protocol lacks specifics related to syringe and pump set‐up.^3^ Our current findings, though not specific to the mixed population of cells in UCB, raise the possibility that the dose delivered in these studies was less than intended. We are not aware of any equivalent studies for UCB derived cells, examining the impact of the various cell infusion components on cell delivery. In light of the observations reported here, we suggest that such studies are merited.

The challenges we have encountered are unique to neonatal intensive care where extremely preterm infants require small infusion volumes delivered slowly. We have been reluctant to concentrate our infusion beyond a postfilter density of 1 million hAECs/mL given an adverse event encountered in our earlier study.^7^ The infant, who became bradycardic and hypoxic following a rapid infusion of hAECs suspended at 2 million cells/mL, likely experienced pulmonary microemboli. The infusion was ceased and the infant recovered quickly. The less concentrated but consequently larger volume infusions need to be delivered slowly (over 60 minutes), which may exacerbate hAEC loss through settling.

The disruption to the delivered volume using the standard method of agitation (removing the syringe from the pump and manually agitating) could have been overcome by determining the volume delivered by reading the syringe volume rather than relying on the pump reading. However, there are drawbacks to this approach. Firstly, disregarding syringe pump readings and relying solely on assessment of syringe volume may not be acceptable in clinical practice and may lead to errors. Furthermore, agitating the syringe at 5‐minute intervals is laborious and not practical clinically. Finally, if infusions were to run for longer or at higher flow rates to deliver the intended volume, the loss of cell density with time observed using the standard protocol may still preclude delivery of the intended dose. To address these concerns, we found an alternate method of agitating the syringe that did not compromise volume delivery. However, as the series of experiments progressed, we found optimizing other parameters actually negated the need for any agitation during a 60‐minute infusion.

An alternate approach may be to leave only the intended hAEC dose in the syringe after priming the IV line, and ensuring dose delivery by infusing the syringe contents. Though appealing in its simplicity, we have reservations regards this approach. We have observed hAECs collecting at the exit point of the vertically positioned syringe (Figure 4). Infusing this likely very dense suspension at the end of the infusion may increase the risk of adverse events such as microemboli.

Another safety concern is flushing the IV line at the end of the infusion. While viable cells accumulate in the IV line, relatively more non‐viable cells accumulate thus reducing the viability of the suspension in the IV line at the end of the infusion to just 65% of the starting viability. Some evidence suggests the immunomodulatory function of some cell therapies appears dependent on apoptosis of the effector cell, leading to the idea that apoptotic cells could be infused.^24^ The efficacy of infusing apoptotic hAECs has not been examined in preclinical models, though purified amnion cell exosomes have shown promise in murine models of idiopathic pulmonary fibrosis.^25^ Further to the unknown efficacy of nonviable hAECs, we are concerned there may be an increased risk of adverse events following infusion of non‐viable cells. The DNA released by nonviable hAECs or hAECs undergoing apoptosis increases cell clumping. If the IV line were to be flushed, infants may receive a denser and potentially “stickier” hAEC suspension increasing the possibility of micro‐emboli.

## 5 SUMMARY

This study highlights a novel, important, and previously overlooked challenge of cell therapy translation in the neonatal population. We have designed a protocol specific to the needs of IV hAEC delivery in extremely preterm infants, contributing both to the integrity of future clinical trials and important safety considerations. This protocol may be applicable to other cell types; however, a similarly rigorous technical evaluation of delivery systems is required before neonatal studies are undertaken.

## CONFLICT OF INTERESTS

P.G.D. declared project and salary support from the Australian National Health and Medical Research Council. R.L. declared employment with Monash University, patent holder with Regenasome Pty Ltd., and Consultant/Advisory role with Meluha Capital. All of the other authors declared no conflicts of interest.

## AUTHOR CONTRIBUTIONS

E.B.: conception and design, collection and/or assembly of data, data analysis and interpretation, manuscript writing, final approval of manuscript. E.W., P.D., S.H.: conception and design, financial support, manuscript writing. A.M., S.J.: conception and design, manuscript writing. R.L.: conception and design, financial support, provision of study material, manuscript writing, final approval of manuscript.

## Data Availability

Data can be made available on reasonable request.
